# Genetic disorders and genetic manipulation at the blood-brain barriers

**DOI:** 10.1186/s12987-023-00428-1

**Published:** 2023-04-20

**Authors:** Richard F. Keep, Hazel C. Jones, Lester R. Drewes

**Affiliations:** 1 Department of Neurosurgery, University of Michigan, R5018 BSRB 109 Zina Pitcher Place, Ann Arbor, MI 48109-2200 USA; 2 Gagle Brook House, Bicester, UK; 3 Department of Biomedical Sciences, University of Minnesota Medical School Duluth, Duluth, MN 55812 USA

The past decade has seen major advances in our understanding of how genetic disorders alter blood-brain barrier (BBB), blood-retinal barrier (BRB) and blood-CSF barrier (BCSFB) function. This has involved studies on patients but also on in vitro models derived from patient samples. Thus, human-induced pluripotent stem cells (iPSCs) used to derive neurovascular unit (NVU) cell types, including brain microvascular endothelial cell (BMEC)-like cells, are an important tool for examining the effects of patient-specific mutations. In addition, gene deletion and overexpression studies in animals and cells are extremely important in dissecting protein- and cell-specific effects on normal BBB/BCSFB function and pathology. They help to provide evidence that BBB and BCSFB dysfunction contributes to neural dysfunction in many neurological conditions (i.e., they are therapeutic targets). Although most studies continue to focus on the impact of gene mutations or deletions, the field of epigenetics has burgeoned providing insights into another level of barrier regulation. While these studies have focused on the effects of genetics/epigenetics on barrier function, it should be noted that normal barrier function is a major impediment to the treatment of genetic disorders impacting the CNS. For example, while enzyme replacement therapies can alleviate the peripheral symptoms of some lysosomal storage disorders, current protein-based therapies do not sufficiently penetrate the BBB to improve CNS pathologies.

To reflect these fields of interest, *Fluids and Barriers of the CNS* launched a thematic series entitled, ‘Genetic Disorders and Genetic Manipulation at the Blood-Brain Barriers’. It provides original research and reviews on these topics as outlined below.

Haploinsufficiency of the BBB glucose transporter, GLUT1, has profound effects on brain development. However, the effects of other genetic mutations on the BBB, the NVU and the BCSFB may be more subtle making it difficult to determine their role in overall neurological dysfunction. Claudin-5 has an essential role in brain endothelial tight junctions and BBB function. Hashimoto et al. [1] review how mutagenesis-based studies have provided insight into claudin-5 function and how sequence alterations may result in a range of neurological and neuropsychiatric disorders. That includes the recent discovery of a missense claudin-5 mutation in patients with alternating hemiplegia of childhood. Zarekiani et al. [2] review recent studies on leukodystrophies, rare monogenic disorders that primarily impact brain white matter but also impact the NVU. They pinpoint the role that new iPSC-derived in vitro models may have in dissecting the effects of leukodystrophies on the NVU. Raut et al. [3] used patient iPSC-derived BMEC-like cells to explore the effects of familial forms of Alzheimer’s disease (mutations in PSEN1 and PSEN2) on the cerebral endothelium. Similarly, Linville et al. [4] examined BMEC-like cells derived from a juvenile Huntington’s disease patient. Both studies found altered endothelial function. This suggests that these diseases directly affect the brain endothelium and may contribute to neurodegeneration. It should be noted that there has been debate over the exact phenotype of iPSC-derived brain endothelial cells as they also express genes associated with epithelial-like properties. Hence, it has been suggested that they be termed BMEC-like cells [5]. As indicated by Linville et al. [6], this also stresses the importance of benchmarking these cells and improving the accuracy of all in vitro BBB models. Zhang et al. [7] have found that inducing expression of the transcription factor Sox18 increases the expression of several BBB-related markers (e.g., P-glycoprotein) in iPSC-derived BMEC-like cells.

Folate transport occurs via folate receptor-alpha, the reduced folate carrier and the proton-coupled folate transporter. Folate is essential for normal neurodevelopment and transporter mutations can cause cerebral folate deficiency. Blood-CSF barrier transport at the choroid plexus plays a central role in brain folate homeostasis but results from Sangha et al. [8] indicate that the arachnoid membrane, another blood-CSF barrier site, may also play an important role.

Brain endothelial mitochondrial dysfunction occurs in multiple neurological conditions (including stroke and Alzheimer’s disease). Lee et al. [9] used TEKCRIF1 knockout mice that have endothelial-specific mitochondrial dysfunction to examine how such dysfunction can lead to BBB disruption. They identified altered NOTCH1 signaling for playing a critical role in this barrier failure.

Xu et al. [10] examined potential causes of hydrocephalus in H-Tx rats. A copy number loss in the chromosome 16p16 region, a region encoding protein tyrosine phosphatase non-receptor type 20 (Ptpn20), was identified as being associated with hydrocephalus. Interestingly, ventriculomegaly also developed in generated Ptpn20 KO mice. This was associated with increased expression of phosphorylated sodium potassium chloride co-transporter-1 (NKCC-1) at the choroid plexus (BCFSB). Phosphorylated NKCC1 has previously been implicated in inducing post-hemorrhagic hydrocephalus [11].

While there is increasing evidence for a role of BBB, NVU and BCSFB dysfunction in many genetic disorders affecting the CNS, it seems likely that neonatal jaundice (hyperbilirubinemia) does not impact BBB and BCSFB function in rats. Blondel et al. [12] examined the Gunn rat that exhibits hyperbilirubinemia because of a mutation in UDP-glucuronosyltransferase UGT1a enzymes and reduced bilirubin catabolism. They found no differences in BBB and BCSFB permeability during development in normo- and hyper-bilirubinemic rats.

Gene deletion/overexpression studies have been used to examine the role of particular proteins at the BBB, NVU, BCSFB and the blood-retinal barriers. Goncalves & Antonetti [13] review crucial findings on barrier function and regulation derived from transgenic animals, including knockouts of specific tight junction proteins. To study the role of low-density lipoprotein receptor-related protein 1 (LRP1) at the BBB, Storck et al. [14] have used a tamoxifen-inducible knockout mouse that specifically targets the cerebral endothelium versus systemic endothelial cells, by employing the solute carrier organic anion transporter Slco1c1 promoter. LRP1 knockdown induced tight junction degradation, reduced BBB integrity and lowered P-glycoprotein levels. Devasani & Yao [15] have reviewed evidence on the role of the ten isoforms of adenylate cyclase (ADCY) in the CNS including at the BBB. Understanding the complexity of G-protein coupled receptor (GPCR)-ADCY signaling, with multiple GPCRs and ADCYs present in multiple cell types in the CNS and NVU, will be aided by more isoform- and cell-specific knockout mice.

He et al. [16] have used a Frizzled-7 CRISPR/Cas9 knockout plasmid and a Frizzled-7 CRISPR activation plasmid to manipulate this important brain endothelial signaling pathway in mice. They found that Frizzled-7 activation can reduce BBB disruption and improve brain edema and neurological function after intracerebral hemorrhage.

Such animal studies have generally been with rodents and particularly mice. It should be noted that Schaffenrath et al. [17] found BBB differences between mouse strains (particularly between wild-derived and long-inbred strains) at the transcriptome level although there was little difference in vascular morphology or permeability to low molecular weight markers. As an alternative to mouse models, zebrafish are becoming increasingly popular for genetic manipulation studies. In zebrafish, Li et al. [18] found that knockout of claudin-5a (but not 5b), an ortholog of mammalian claudin-5, caused BBB disruption, severe brain edema and brain herniation during development.

Genetic manipulations can be performed both in vitro and in vivo. Baumann et al. [19] found that astrocyte hypoxia-inducible factor-1 (HIF-1) was not important in hypoxia-induced endothelial disruption in *vitro* using HIF-1 siRNA. That group then expanded their studies to in vivo using cell-specific inducible knockouts [20] and found that pericyte, but not astrocyte, HIF-1 depletion reduced hypoxia-induced endothelial barrier permeability. There can be concerns when inducing expression of proteins that abnormal cellular distributions may occur. Gericke et al. [21] have examined this for P-glycoprotein in brain endothelial cell lines in vitro.

Within the NVU, the importance of the extracellular matrix, including proteins such as laminin, has been underappreciated. This is changing thanks largely to the results of gene deletion studies showing marked effects on BBB function. Halder et al. [22] review studies on the role of different laminin isoforms and their integrin receptors on BBB development, maturation and stability.

While genetic disorders may directly impact the BBB/NVU, they may also make the BBB/NVU susceptible to other injuries. Thus, Clark et al. [23] found that young transgenic mice overexpressing human amyloid precursor protein (APP) and presenilin 1, a model of Alzheimer’s disease, when exposed to blast-induced traumatic brain injury, had lowered perivascular aquaporin-4 and chronic capillary proliferation.

Compared to the BBB/NVU, there has been a relative paucity of studies using genetic manipulations of the choroid plexus epithelium. Jang & Lehtinen [24] review current methodologies for performing such studies in vivo using genetic models and viral gene delivery, and also discuss future approaches.

Epigenetics is a growing field of barrier research. Ihezie et al. [25] review the role different types of epigenetic mechanisms (DNA methylation, histone modification, non-coding RNAs) in regulating the BBB and potential therapeutic interventions targeting those mechanisms. Phillips et al. [26] examined the role of changes in DNA methylation (the methylome) in BBB recovery after ischemic stroke in mice and how this response is altered with aging. Importantly, they correlated changes in the methylome with the transcriptome, and described how methylation status can lead to incomplete BBB recovery which may worsen stroke outcome and enhance the chances of recurrent stroke. While DNA methylation is a key epigenetic regulator, the family of ten-eleven translocation (TET) methylcytosine dioxygenases can demethylate DNA. Wang et al. [27] have used knockout mice and siRNA mediated knockdown of TET2 to examine the role of such demethylation in regulating the tight junction protein, ZO-1. Sun et al. [28] review the role of non-coding RNAs (including microRNAs, long non-coding RNAs and circular RNAs) in BBB regulation and in a range of neurological diseases.

The BBB remains an enormous obstacle to treating neurological diseases, including genetic disorders. While there has been great growth in the field of biologics to treat human diseases, those agents very seldom cross the BBB. For example, proteins normally have very low BBB permeabilities and much research has focused on enhancing uptake. Sahin et al. [29] discuss this in relation to treating mucopolysaccharidoses. The systemic effects of these lysosomal storage disorders can be ameliorated with enzyme replacement therapy, but those enzymes do not cross the BBB. Sahin et al. [29] describe current methodologies for enhancing delivery of enzymes to brain. One approach to bypass the BBB is direct injection into the CSF. However, Kahni et al. [30] using modeling suggest that varying injection parameters and differences in patient physiology can alter drug delivery (including gene therapy) via this route.

In conclusion, this thematic series shows the breadth of ongoing studies examining the effects of genetic disorders on the BBB/NVU, using genetic manipulations to understand BBB, BRB and BCSFB physiology and pathology, exploring epigenetic regulation, and devising ways to allow therapeutics to enter the brain to treat CNS genetic disorders. It is hoped that such investigations and our greater understanding will help develop treatments for CNS genetic disorders and other neurological conditions.

## Data Availability

Not applicable.
